# Natural oil blend formulation as an anti-African swine fever virus agent in *in vitro* primary porcine alveolar macrophage culture

**DOI:** 10.14202/vetworld.2021.794-802

**Published:** 2021-03-30

**Authors:** Quang Lam Truong, Lan Thi Nguyen, Haig Yousef Babikian, Rajeev Kumar Jha, Hoa Thi Nguyen, Thanh Long To

**Affiliations:** 1 Key Laboratory of Veterinary Biotechnology, Faculty of Veterinary Medicine, Vietnam National University of Agriculture, Hanoi, Vietnam; 2 Department of Research and Development, PT. Rhea Natural Sciences, Jakarta, Indonesia

**Keywords:** African swine fever virus, *in vitro* trials, natural oil blend formulation, primary porcine alveolar macrophages cells

## Abstract

**Background and Aim:**

African swine fever is one of the severe pathogens of swine. It has a significant impact on production and economics. So far, there are no known remedies, such as vaccines or drugs, reported working successfully. In the present study, the natural oil blend formulation’s (NOBF) efficacy was evaluated against ASFV *in vitr*o using porcine alveolar macrophages (PAMs) cells of swine.

**Materials and Methods:**

The capacity of NOBF against the ASFV was tested *in vitro*. The NOBF combines *Eucalyptus globulus*, *Pinus sylvestris*, and *Lavandula latifolia*. We used a 2-fold serial dilution to test the NOBF formulation dose, that is, 10^5^ HAD50/mL, against purified lethal dose of African swine in primary PAMs cells of swine. The PAM cells survival, real-time polymerase chain reaction (PCR) test, and hemadsorption (HAD) observation were performed to check the NOBF efficacy against ASFV.

**Results:**

The *in vitro* trial results demonstrated that NOBF up to dilution 13 or 0.000625 mL deactivates the lethal dose 10^5^ HAD50 of ASFV. There was no HAD (Rosetta formation) up to dilution 12 or 0.00125 mL of NOBF. The Ct value obtained by running real-time PCR of the NOBF group at 96 h post-infection was the same as the initial value or lower (25), whereas the Ct value of positive controls increased several folds (17.84).

**Conclusion:**

The *in vitro* trial demonstrated that NOBF could deactivate the ASFV. The NOBF has the potential to act as anti-ASFV agent in the field. The next step is to conduct *in vivo* level trial to determine its efficacy.

## Introduction

African swine fever virus (ASFV) reported as deadly for pigs. It is listed as a “notifiable disease” by the OIE due to high illness rates and a high mortality rate, up to 100%, and substantial financial losses [1-3]. Further spread of ASF to China has had disastrous consequences, especially instead of the fact that China contains more than half of the world’s pig population [4]. To date, as far as Vietnam is concerned, ASF has appeared in all 63 provinces of Vietnam, has destroyed more than 5.6 million pigs (more than 20% of total pigs), has decreased pork production by 8.3%, and has affected mainly small-scale farms [5-8].

The typical signs and symptoms of ASF are high fever, decreased appetite and weakness, difficulty in standing, red or blue blotches on the skin (particularly around ears and snout), and, especially in sows, the symptoms of miscarriage, stillbirths, and weak litters can occur [9,10]. Like, diarrhea, vomiting, and difficulty breathing or coughing, the symptoms can also occur with the disease [9]. ASFV is a large, enveloped and structurally complex DNA virus with the *Asfarviridae* family’s icosahedral morphology. The virus can persist for a long time in the environment, carcasses, and various swine products. The vectors and carriers of the ASF virus are warthogs (*Phacochoerus africanus*), bush pigs (*Potamochoerus porcus* and *Potamochoerus larvatus*), and soft ticks (*Ornithodoros moubata*) [4] in which the virus is transmitted trans-staidly and through transovarial routes [9].

The role of natural oils as antiviral components is well known. As a standardized compound, natural products are significant components with antiviral properties [11]. A formulation was developed by blending three natural oils, *Eucalyptus globulus*, *Pinus sylvestris*, and *Lavandula latifolia*, with antiviral properties. Cineole, the significant component of eucalyptus oil, has potent anti-inflammatory and anti-microbial properties [12]. Cineole is well known to treat the respiratory tract’s primary viral infections [13,14]. Linalool, a significant lavender oil component, has shown antiviral activities [15-17]. Isobornyl acetate extracted from pine oil has anti-microbial properties [14].

In the present study, the natural oil blend formulation’s (NOBF) efficacy was evaluated against ASFV using porcine alveolar macrophage (PAM) cells of swine in an *in vitro* medium.

## Materials and Methods

### Ethical approval

The protocols for pig euthanization and lung collection for isolation of primary PAM cells from healthy pigs and *in vitr*o study were approved by the Animal Welfare and Ethics Committee of Vietnam National University of Agriculture, Vietnam.

### Study location and period

In this trial, we selected 7-10-week-old healthy pigs negative for ASFV, PCV2, CSFV, PRRSV, and negative for ASFV Ab for isolation of PAM cells. The animals were housed and used in an isolated area in the Biosecurity Animal Facility Centre of the Vietnam National University of Agriculture (VNUA), Hanoi, Vietnam. The study was conducted from October 2019 to January 2020.

### NOBF development

A formulation was developed by mixing an essential oil blend using three oils, *E. globulus*, *P. sylvestris*, and *L. latifolia*, in a determined concentration. The oils, *E. globulus*, *P. sylvestris*, and *L. latifolia*, obtained from the vendors who comply with the strictest industry practices: Demeter Agro Research and Improvements Pty Ltd., Australia, New Directions Australia Pty Ltd., Australia, and Australian Botanical Products Pty Ltd, Australia. Each natural oil is obtained through the steam distillation process and should undergo thorough checking for the quality and chemical compositions based on European Pharmacopeia. After the natural oils are declared to pass the quality checking, the mixture of the NOBF conducted with the following sequence and percentage: *E. globulus*, *P. sylvestris*, and *L. latifolia* are added in equal quantities to form the NOBF.

We followed a 2-fold dilution procedure to obtain the optimum dose of the application. The NOBF was serially diluted from 2.5 mL to 0.000078 mL (dilution 1 to dilution 16) to perform the *in vitro* trials.

### Toxicity test of NOBF

The formulation was pre-tested on animals for toxicity and tolerance level. The toxicity level of NOBF was tested at two levels. The first level test was performed in the PAM cells, whereas the tolerance level of NOBF was tested in live pigs. The NOBF in 16 double-fold dilutions of 2.5 mL-0.000078 mL was mixed with the PAM cells and the ASFV. The cell viability was tested under a microscope at every 24 h interval.

### Active ingredient identification in NOBF

The gas chromatography technique was used to extract and identify the active ingredients with anti-ASFV properties. Gas Agilent 6890 and 7890 Gas Chromatography-Flame Ionization Detector Shimadzu GC 2014 analysis was conducted at the Faculty of Pharmacy Laboratory at the University of Indonesia. The details of the standards used in this analysis are as follows:

**a) Standard 1,8-Cineole**


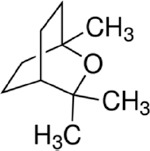


Formula: C_10_H_18_O

Molecular weight: 154.25 g/mol

CAS-No.: 470-82-6

Product number: C80601

Brand: Sigma-Aldrich, USA

**b) Standard linalool**


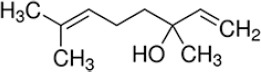


Formula: C_10_H_18_O

Molecular weight: 154.25 g/mol

CAS-No.: 126-91-0

EC-No.: 204-811-2

Product NUMBER: 74856

Brand: Sigma-Aldrich

**c) Standard isobornyl acetate**


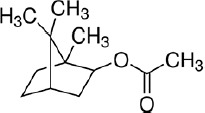


Formula: C_12_H_20_O_2_

Molecular weight: 196.29 g/mol

CAS-No.: 125-12-2

EC-No.: 204-727-6

Product number: W216003

Brand: Sigma-Aldrich

### Primary PAM cells

Our team collected the primary PAMs from 7-week-old healthy pigs (negative for ASFV, PCV2, CSFV, and PRRSV). We maintained the cells in the growth medium, including an RPMI 1640 medium (Gibco, USA) supplemented with 10% fetal calf serum (FCS; Gibco) and 1% penicillin-streptomycin solution (Gibco) at 37°C with 5% CO_2_. We also prepared red blood cells from EDTA-treated swine blood using Percoll (GE Healthcare, USA) and kept it in an RPMI 1640 medium (Gibco), 1% penicillin-streptomycin solution, and maintained it at 4^o^C until use.

### ASFV preparation

The VNUA-ASFV-L01/HN/04/19 virus strain was isolated from a pig with apparent symptoms in Thai Binh Province, Vietnam. The virus strain was purified and quantified in the molecular biology laboratory of VNUA, Vietnam. The lethal and sublethal doses were optimized in the controlled conditions. The ASFV strain VNUA01/04.2019 was adapted to grow in PAMs and further passaged in PAMs. The stock used in the present study was obtained after the 15^th^ passage. Briefly, the PAMs were infected at a multiplicity of infection of 0.1 with VNUA-ASFV-L01/HN/04/19 in the growth medium, including an RPMI 1640 medium supplemented with 10% FCS and 1% penicillin-streptomycin solution. We added to each well the maintenance medium containing an RPMI 1640 medium supplemented with 5% FCS, 1% penicillin-streptomycin solution, and 2% suspension of red blood cells. The team performed the ASFV titration on the PAMs cultures in 96-well plates. The presence of ASFV was assessed by hemadsorption (HAD). The observation of HAD was for 5 days, and the calculation of 50% HAD observation (HAD50) was performed using the method described by Reed *et al*. [18] and King *et a*l. [19].

### Antiviral activity of NOBF using an *in vitro* medium

PAMs were grown on 48-well tissue culture plates using a growth medium, including an RPMI 1640 medium supplemented with 10% FCS and 1% penicillin-streptomycin solution. The NOBF was serially 2-fold diluted (Table-1) in a warmed RPMI 1640 to prepare the working stocks after 3 h of incubation of serially 2-fold diluted NOBF with VNUA-ASFV-L01/HN/05/19.

**Table-1:** Natural oil blend formulation (NOBF) mixed with RPMI and African swine fever virus (ASFV) in serial fold 2 dilution.

Dilution	ASF Virus (mL)	NOBF (mL)	RPMI (mL)	NOBF (%)	NOBF (ppm)
1	5	2.5	2.5	25	250,000
2	5	1.25	3.75	13	125000
3	5	0.625	4.375	6.30	62500
4	5	0.3125	4.6875	3.10	31250
5	5	0.156	4.998	1.56	15600
6	5	0.08	4.92	0.80	8000
7	5	0.04	4.96	0.40	4000
8	5	0.02	4.98	0.20	2000
9	5	0.01	4.99	0.10	1000
10	5	0.005	4.995	0.05	500
11	5	0.0025	4.9975	0.03	250
12	5	0.00125	4.99875	0.01	125
13	5	0.000625	4.999375	0.01	62.5
14	5	0.000312	4.999688	0.00	31.25
15	5	0.000156	4.999844	0.00	15.625
16	5	0.000078	4.999921	0.00	7.812
Positive Control	5	-	5	-	-
Negative Control	(buffer 5 ml)	-	5	-	-

The 5 mL of VNUA-ASFV-L01/HN/05/19 mixed in each tube except negative control. The NOBF was serially 2-fold diluted up to 7.8 ppm. NOBF=Natural oil blend formulation

**Figure-1:**
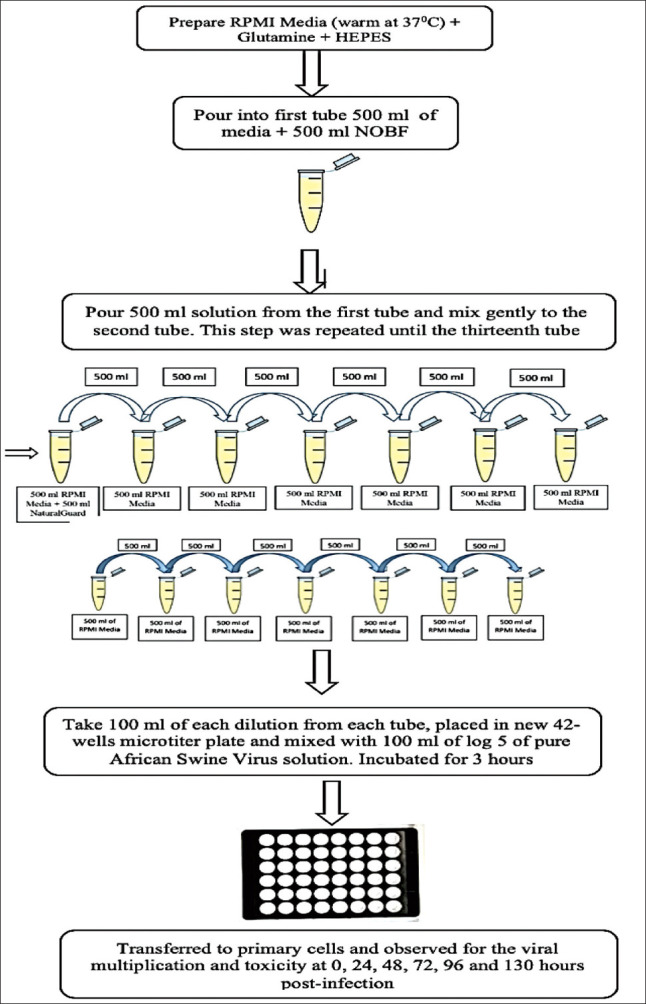
Diagrammatic illustration of *in vitro* trial steps preparation. RPMI media, natural oil blend formulation, and African swine fever virus prepared and mixed using 2-fold dilution method. No virus was mixed in negative control. Microscopic observation was made at 0 h, 24 h, 48 h, 72 h, 96 h, and 130 h intervals.

The ASF virus dose 10^5^ HAD50 in the ratio of 1:1, duplicate cultures were infected with the corresponding virus in a diluted volume of a medium containing the NOBF at 37°C in an atmosphere of 5% CO_2_ for 1 h (Table-1 and Figure-1). The culture was added to the maintenance medium and incubated until a massive cytopathic effect, such as HAD or rosette formation. Rosette formation was observed daily by an inverted microscope for 4-5 days. After four freeze-thaw cycles, the supernatants were assessed for the ASFV virus by real-time polymerase chain reaction (PCR) using the OIE manual’s recommendations described by Juürgen *et al*. [20].

## Results

### The active ingredient NOBF

**Figure-2:**
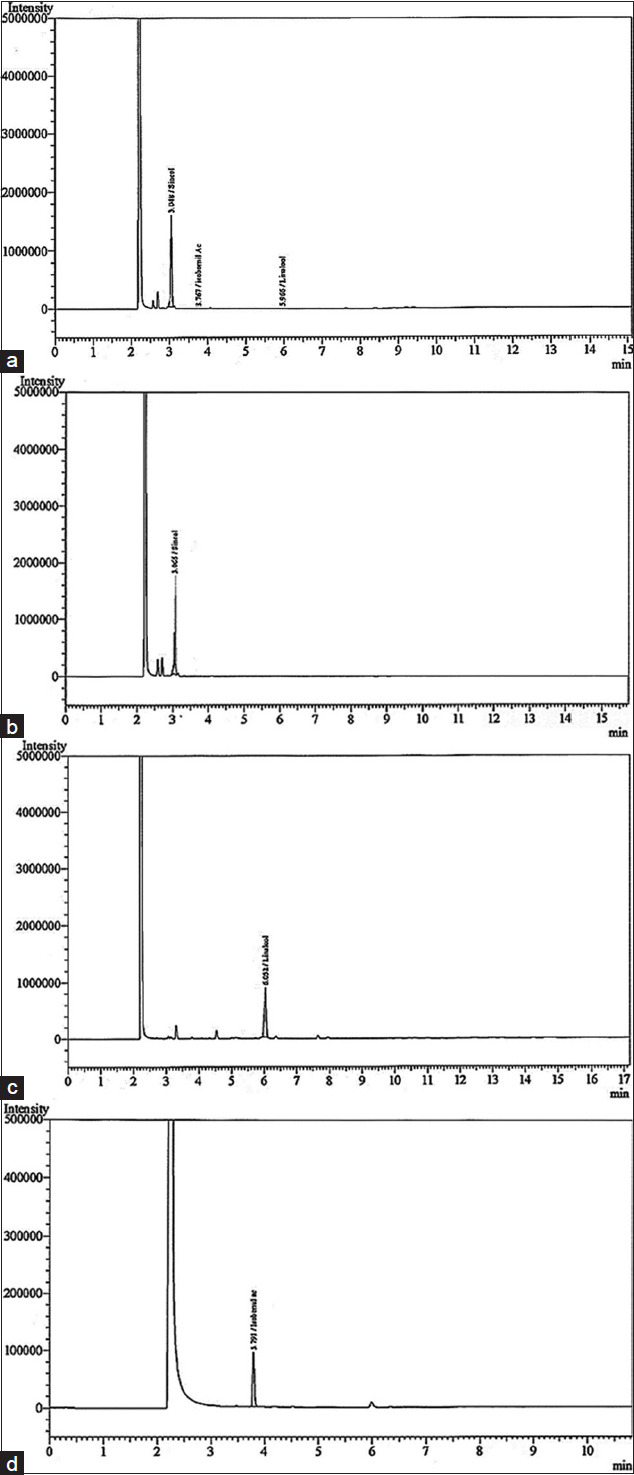
Gas chromatography-flame ionization detector chromatogram of natural oil blend formulation (NOBF). (a) Chromatogram of complete NOBF compound’s peak, that is, cineole, linalool, and isobornyl acetate, (b) Chromatogram of cineole from NOBF, (c) chromatogram of linalool from NOBF, and (d) chromatogram of isobornyl acetate from NOBF.

The gas chromatography technique measures the active ingredients present in the blended oil. By comparing with chromatographs of pure standards, GC analysis identified the presence of antiviral compounds, namely, (A) cineole, (B) linalool, and (C) isobornyl acetate in the NOBF (Figure-2). The result shows that cineole was present at a retention time of 3.048 min with a relative percentage area of peak around 99.4%, making it the primary compound in the NOBF. The second compound identified is linalool at a retention time of 5.966 min and a relative percentage peak around 0.31%. The minor compound identified is isobornyl acetate at a retention time of 3.767 and a relative percentage area of peak around 0.28% (Table-2).

**Table-2:** Gas chromatography-flame ionization detector chromatogram of natural oil blend formulation in tabular form with complete details of measurement and analysis.

Peak No.	Name	Ret. time	Area	Height	HETP	Resolution	Tailing factor	k᾿	Separation
1	Cineole	3.048	3516085	11587085	0.000	0.000	0.831	0.000	0.000
2	Linalool	5.966	11108	4328	0.000	12.943	0.840	0.236	0.000
3	Isobornyl acetate	3.767	10037	2854	0.000	30.333	0.000	0.957	4.057
Total			3537230	11594267					

### Toxicity test and efficacy of NOBF in PAMs culture against ASFV

**Figure-3:**
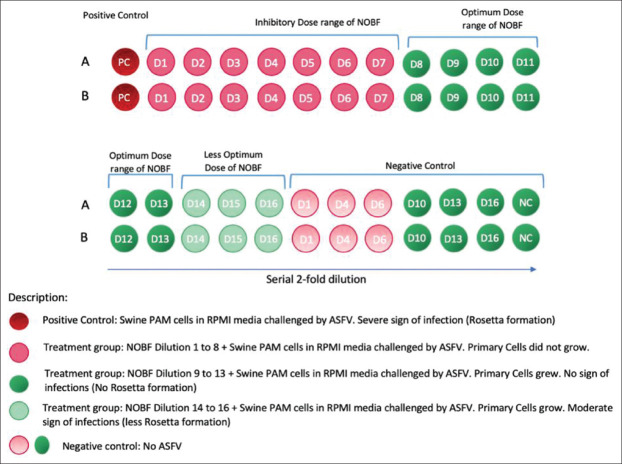
The illustration shows the result outcome of *in vitro* test. The inhibitory dose of the natural oil blend formulation (NOBF) was observed from dilution 1-7. The optimum and effective dose of NOBF against African swine fever virus (ASFV) was observed from dilutions 8-13. The less effective dose started from dilutions 14. The different dilutions of negative control (without ASFV) indicated the inhibitory and not-inhibitory doses of NOBF on porcine alveolar macrophages cells.

**Figure-4:**
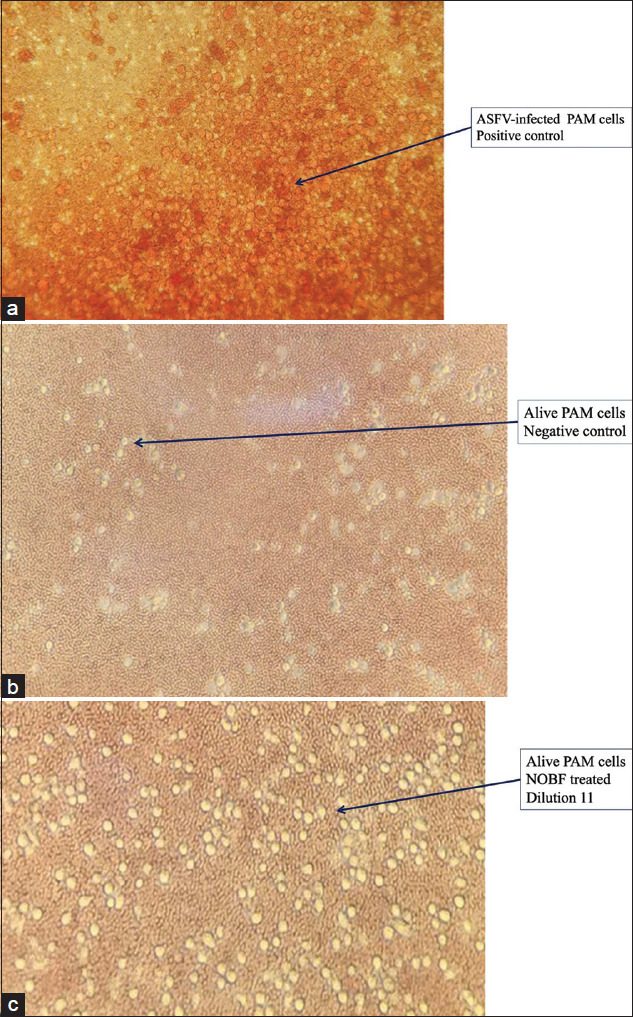
(a) Representative microscopic images of positive control, that is, porcine alveolar macrophages (PAMs) cells inoculated with African swine fever virus. (b) Representative microscopic images of negative control, that is, PAM cells not inoculated with African swine fever virus. (c) Representative microscopic images of PAM cells inoculated with African swine fever virus treated in dilution 11or 250 ppm of natural oil blend formulation for 130 h. (c) Images were taken on Leica DM IL LED microscope at 200×

The toxicity of the NOBF in PAMs cultures was tested in PAM cells along with the trial. PAM cell death was recorded up to dilution six at 36 h of observation. Cell death occurred in dilution seven wells at 48 h and dilution eight at 72 h of culture. No PAM cells died in dilution nine and onward until the last observation at 120 h. The obtained results show that the NOBF in dilutions 1-6, that is, up to 0.08 mL, inhibited the growth of PAM cells (Figures-3 and 4).

The detailed stepwise observation at 96 h post-infection occurred as follows:

*In vitro* observation showed that the NOBF up to dilution 13 or 0.000625 mL could inhibit or degenerate ASFV at a titer of 10^5^ HAD50 in the PAMs culture (Table-1). No HAD or rosette was observed up to 130 h post-infection (Figure-3 and 4) whereas positive controls showed a large number of rosettes formation.

### Real-time PCR analysis of *in vitro* trial

After four freeze-thaw cycles of PAMs, the supernatants were collected and applied for total DNA extraction, then used for real-time PCR [20]. The real-time PCR results (Ct value) indicated that the virulent ASFV strain could not replicate or was denatured in the PAMs cultures (Table-3). No difference in Ct value obtained in the initial ASFV input control compared to the natural oil blend group at dilutions 10, 11, and 12 after 130 h post-infection in PAMs (Table-3). Remarkably, the difference in the natural oil blend group’s Ct value was statistically significant compared with the ASFV-positive control group (Table-3).

**Table-3:** Real-time PCR quantification of ASFV replication in treatment groups *in vitro.*

No.	Groups	Dilution	HAD^β^/real-time PCR	

			Rosetta formation	Real-time PCR Ct value (mean)
1	Group 1: Natural oil blend formulation	10	No	25.96
2		11	No	25.89
3		12	No	25.16
4		13	No	23.97
5	Group 2: Positive control		Yes	17.84
6	Group 3: Negative control		No	NA
7	Input control of ASFV^α^			25.74

α Initial ASFV dose that is used to apply all groups except negative control group. African swine fever virus. ASFV=African swine fever virus, PCR=Polymerase chain reaction

## Discussion

African swine fever is a highly contagious fatal acute hemorrhagic viral disease of pigs that currently have no treatment or vaccination protocol. It threatens the pig industry worldwide. For farmers, managing recent outbreaks of infectious viral diseases remain a significant worldwide problem, and there is a need to find substances with intracellular and extracellular antiviral properties [21-26]. The ASFV precisely activates the Ataxia Telangiectasia Mutated Rad-3-related (ATR) pathway in ASFV-infected swine monocyte-derived macrophages (MDMs) in the early phase of infection, most probably because the ASFV genome is recognized as foreign DNA [27]. They also detected morphological changes of promyelocytic leukemia nuclear bodies, nuclear speckles, and Cajal bodies found in ASFV-infected swine MDMS. It suggests the process of viral modulation of cellular antiviral responses and cellular transcription. It was demonstrated that *in vitro* inhibition of ASFV-topoisomerase II disrupts viral replications, contributing to natural strategies for vaccine candidate development [28].

The *in vitro* level trials using the NOBF were conducted to evaluate its efficacy against ASFV. The natural oil blend had antiviral properties and deactivated the virus [29-31]. The antiviral activity of all the natural oils tested could be demonstrated for the enveloped viruses. Lavender natural oil consists primarily of monoterpenoids and sesquiterpenoids and linalool dominate, with moderate levels of lavandulyl acetate, terpinen-4-ol, and lavandulol. 1,8-cineole and camphor are also present in low to moderate qualities. Linalol has anti-microbial, anti-inflammatory, and mood alleviating effects [32-34]. Pine oil consists mainly of alpha-terpineol or cyclic terpene alcohols and isobornyl acetate. Pine oil is a phenolic disinfectant that is mildly antiseptic and has ­anti-fungal, ­antibacterial, and antiviral properties due to isobornyl acetate [33,34]. Eucalyptus oil has a history of full application as a pharmaceutical, antiseptic, repellent, flavoring, fragrance, and industrial use. Eucalyptus oil has antibacterial, antiviral, and anti-inflammatory effects. The pre-clinical results also show that eucalyptus oil stimulates the innate ­cell-mediated immune response by its effects on the phagocytic ability of human MDMs [31-37]. Cineole present in eucalyptus oil shows potential antiviral activity against herpesvirus and yellow fever virus. Its activity has also been established against viral envelope structures [34,35]. The natural oils affect the viral envelope, which is necessary for adsorption or entry into host cells. In particular, monoterpenes have shown increased cell membrane fluidity and permeability, altering membrane proteins’ order [38-41].

The quantity log 5 of the VNUA-ASFV-L01/HN/04/19 virus strain ASFV is considered the lethal dose that can kill the pigs in 7-10 days intramuscular challenge. The evaluation of *in vitro* antiviral activities of natural substances is based mainly on the inhibition of cytopathic effects, the reduction or inhibition of plaque formation, and the reduction in the virus yield [21].

The PAM cells were isolated from the pathogen-free clean piglets. The characteristic feature of the cells infected with ASFV wildtype is the ability to adsorb swine erythrocytes (haemadsorption - HAD) on its surface [40]. This feature was successfully exploited to differentiate the ASF virus from other agents that produce diseases with symptoms that are likely to be confused with those observed in ASF and develop specific ASF virus titration [42]. Erythrocyte rosettes formation around infected blood swine monocytes is a characteristic feature of ASFV-infected cells [43,44]. It is considered the standard HAD test for many ASFV isolates

The real-time PCR results showed no growth of the virus in the NOBF groups indicated from the similar Ct value. In comparison, the Ct value of positive control reduced, which indicated the spontaneous increase in viral copy number [20].

The obtained results showed the way in the process of anti-ASFV component development. Hence, the anti-ASFV developed components, either ­live-attenuated vaccine or recombinant vaccine, have not shown efficacy in the field [45-49]. The immediate next step would be to evaluate and optimize the efficacy of NOBF against ASFV in the pig.

## Conclusion

The present study highlights the role of natural oil extracts in virus eradication. In this study, we try to demonstrate that the NOBF can degenerate the ASFV in PAM cells medium. The trial outcome is summarized in the following way: The NOBF application deactivated the ASF virus at the lowest concentration of 0.000635 mL. As a continuation of the study, the next step is to conduct *in vivo* trials to optimize the dose and delivery route of the NOBF and to establish it as an anti-ASFV candidate.

## Authors’ Contributions

QLT, HYB, and RKJ contributed to the study conception. HTN contributed to sample analysis. QLT, HYB, and RKJ designed and conducted the experiments. QLT, HYB, and RKJ analyzed the data. HYB and RKJ drafted the manuscript. LTN and TLT edited the manuscript. All authors read and approved the final manuscript.
